# Genetic Association of Circulating Proteins and Gene Transcripts With Spontaneous Coronary Artery Dissection

**DOI:** 10.1161/CIRCGEN.125.005330

**Published:** 2026-01-29

**Authors:** Maddalena Ardissino, Alec P. Morley, Buu Truong, Art Schuermans, Rohin K. Reddy, Martina Milani, Alice Sacco, James S. Ware, Stephen Burgess, Brian P. Halliday, Thong Huy Cao, Paulene A. Quinn, Leong L. Ng, Ania A. Baranowska-Clarke, Pradeep Natarajan, Adam S. Butterworth, Michael C. Honigberg, Kypros Nicolaides, Thomas Webb, David Adlam, Antonio de Marvao

**Affiliations:** Medical Research Council Laboratory of Medical Sciences, Imperial College London, United Kingdom (M.A., J.S.W., A.M.).; National Heart and Lung Institute, Imperial College London, United Kingdom (R.K.R., J.S.W., B.P.H.).; British Heart Foundation Cardiovascular Epidemiology Unit, Department of Public Health and Primary Care (M.A., A.S.B.); Department of Medicine, School of Clinical Medicine (A.P.M.); Gonville and Caius College (A.P.M.), University of Cambridge, United Kingdom.; Medical Research Council Biostatistics Unit (S.B.), University of Cambridge, United Kingdom.; Victor Phillip Dahdaleh Heart & Lung Research Institute (A.S.B.), University of Cambridge, United Kingdom.; British Heart Foundation Centre of Research Excellence (A.S.B.), University of Cambridge, United Kingdom.; Health Data Research UK Cambridge, Wellcome Genome Campus (A.S.B.), University of Cambridge, United Kingdom.; National Institute for Health Research Blood and Transplant Research Unit in Donor Health and Genomics (A.S.B.), University of Cambridge, United Kingdom.; Program in Medical & Population Genetics & Cardiovascular Disease Initiative, Broad Institute of Harvard & MIT, Cambridge, MA (B.T., A. Schuermans, P.N., M.C.H.).; Center for Genomic Medicine & Cardiovascular Research Center, Massachusetts General Hospital (B.T., A. Schuermans, P.N., M.C.H.), Harvard Medical School, Boston, MA.; Department of Medicine (P.N., M.C.H.), Harvard Medical School, Boston, MA.; Faculty of Medicine, KU Leuven, Belgium (A. Schuermans).; Nuffield Department of Population Health, University of Oxford, United Kingdom (R.K.R.).; Cardiology Department, Alessandro Manzoni Hospital, ASST Lecco, Italy (M.M.).; Cardiac Intensive Care Unit, “De Gasperis Cardio Centre,” ASST Grande Ospedale Metropolitano Niguarda, Milan, Italy (A. Sacco).; Royal Brompton & Harefield Hospitals, Guy’s and St Thomas’ NHS Foundation Trust, London, United Kingdom (J.S.W., B.P.H.).; Imperial College Healthcare NHS Trust, London, United Kingdom (J.S.W.).; Department of Cardiovascular Sciences, College of Life Sciences (T.H.C., P.A.Q., L.L.N., A.A.B.-C., T.W., D.A.), University of Leicester, United Kingdom.; Leicester van Geest Multi-OMICS Facility (T.H.C., P.A.Q., L.L.N.), University of Leicester, United Kingdom.; British Heart Foundation Centre of Research Excellence (T.H.C., P.A.Q., L.L.N., D.A.), University of Leicester, United Kingdom.; NIHR Leicester Biomedical Research Centre, University Hospitals of Leicester NHS Trust, Glenfield Hospital, United Kingdom (T.H.C., L.L.N., T.W., D.A.).; Department of Women & Children’s Health (K.N., A.M.), King’s College London, United Kingdom.; Fetal Medicine Research Institute (K.N., A.M.), King’s College London, United Kingdom.; British Heart Foundation Centre of Research Excellence, School of Cardiovascular & Metabolic Medicine & Sciences (A.M.), King’s College London, United Kingdom.

**Keywords:** coronary vessels, extracellular matrix proteins, genetics, Mendelian randomization analysis, pregnancy

## Abstract

**BACKGROUND:**

Spontaneous coronary artery dissection (SCAD) is an uncommon cause of myocardial infarction that disproportionately affects women, particularly during pregnancy and the peripartum period. Limited understanding of its underlying pathophysiology hinders the development of effective preventive and therapeutic strategies.

**METHODS:**

This study investigated associations between genetically predicted circulating proteins and tissue-specific RNA levels with genetically predicted SCAD risk using Mendelian randomization and Bayesian colocalization. Genetic scores for >1500 circulating proteins were derived from the UK Biobank (N=34 557) and deCODE (N=35 559). Scores for 13 848 gene transcripts in arterial and fibroblast tissues were generated from Genotype-Tissue Expression data. Associations between these scores and SCAD were assessed in a genome-wide association study meta-analysis of 1917 individuals with SCAD and 9292 controls. Findings were validated in vitro using mass spectrometry-based proteomic analysis of extracellular vesicles from 50 patients with SCAD and 50 healthy controls.

**RESULTS:**

Genetic associations of 4 circulating proteins with SCAD (AFAP1 [actin filament–associated protein 1], ECM1 [extracellular matrix protein 1], SPON1 [spondin 1], and STAT6 [signal transducer and activator of transcription 6]) were identified. Two were supported by gene expression data (AFAP1 and ECM1), and one by tissue-specific Bayesian colocalization analyses (ECM1). Protein interaction mapping identified potential shared pathways through the JAK-STAT (Janus kinases and signal transducers and activators of transcription) signaling pathway and inflammatory regulation. Mass spectrometry-based proteomic analysis demonstrated that ECM1 was significantly upregulated in SCAD cases versus controls.

**CONCLUSIONS:**

Integrative analysis of proteomic, transcriptomic, and experimental data revealed 4 circulating proteins genetically associated with SCAD risk, with ECM1 emerging as a key protein with a likely causal role in SCAD pathogenesis. These findings highlight biological pathways for mechanistic studies and protein targets for potential therapeutic interventions.

Spontaneous coronary artery dissection (SCAD) is characterized by the spontaneous formation of an intramural hematoma within the wall of a coronary artery, which can lead to a tear in the intimal layer, formation of a false lumen, compression of the true lumen, and ultimately, myocardial ischemia or infarction. Unlike atherosclerotic causes of acute coronary syndrome, SCAD predominantly affects young women, who account for nearly 88% of cases.^1^ Although SCAD represents <1% to 4% of acute coronary syndrome presentations overall,^2^ it is responsible for ≈25% of acute coronary syndrome cases in women under the age of 50 years,^3^ and is the most common cause of myocardial infarction during pregnancy (43%^4^). There is growing evidence of a genetic susceptibility to SCAD beyond the association with pathogenic variants in inherited connective tissue disorders and aortopathies, such as Marfan, Loeys-Dietz, and Ehlers-Danlos.^5,6^ Despite its significant clinical impact, the underlying pathophysiological mechanisms of SCAD among people without connective tissue disorder, which represent the majority of SCAD cases,^7^ remain poorly understood,^8^ limiting the development of targeted treatments. Current approaches for the prevention and treatment of SCAD are often adapted from atherosclerotic acute coronary syndrome treatments rather than being tailored to its unique pathology, potentially leading to lower efficacy and more complications.^1,9^

Discovering effective drug targets often involves identifying causal proteins with a fundamental role in disease pathways.^10^ However, pinpointing causal proteins in diseases like SCAD is challenging given the complex interactions across biological systems. Recent advances in proteomic and transcriptomic profiling have generated large repositories of genetic variants that influence protein levels and gene expression across various tissues. Leveraging such data enables the identification of key proteins and mediators within the causal disease pathway, which provides the advantage of streamlining discovery and efficiency of pharmaceutical development by providing rationale for early preclinical investigations.^11^

Mendelian randomization (MR) is a genetic epidemiological approach that harnesses the random allocations of genetic alleles at conception to help determine whether certain risk factors have a causal relevance to a disease process. Such variation enables MR studies to be analogous to a randomized control trial, which is particularly helpful in rare diseases, where it can be difficult to recruit sufficient numbers to investigational trials, provided sufficient genetic data is available for analysis. Proteomics-based MR exploits the natural genetic variation that influences protein concentrations to predict the consequences of perturbation of these proteins on an outcome. This reduces common epidemiological challenges, such as confounding and reverse causation, providing evidence to support causality.^12^ Nonhuman disease models, often used for lead-identification during preclinical pharmaceutical trials, do not necessarily mirror human disease phenotypes, and this can hamper the drug discovery process. This makes MR valuable in this context, as results are based on human genetic evidence that, when combined with other types of evidence, can facilitate more targeted clinical trials and improve the likelihood of regulatory approval success.^13,14^ Furthermore, integrative approaches with genome-wide association studies (GWAS) and proteome/transcriptome MR analyses can provide greater understanding of complex causal pathways, when compared with GWAS alone.^15^

In this study, we performed a comprehensive proteome-wide genetic analysis to evaluate the causal relevance of >1500 circulating genetically predicted proteins for the genetic risk of SCAD across 2 different proteomic platforms. We broadened and corroborated these analyses by leveraging transcriptomic data on the expression of >13 500 genes across arterial tissue and cultured fibroblasts, which are tissues of key relevance to SCAD. We then annotated key proteins associated with SCAD for druggability and performed a phenome scan to assess for other known protein-trait associations. Finally, we validated our findings experimentally by conducting a mass spectrometry-based proteomic analysis on a cohort comprising 50 patients with SCAD and 50 healthy controls, examining the differential expression of candidate proteins identified as significant in the genetic analyses.

## Methods

Summary-level genetic association data sets were obtained from public repositories (GWAS Catalog; GTEx [Genotype-Tissue Expression] v8) and proteomic protein quantitative trait loci (pQTL) resources (deCODE SOMAscan v4 and UKB [UK Biobank] Olink Explore), as cited.^16–20^ Processed data underlying figures/tables are in the Supplemental Material, and individual-level validation proteomics are available as deidentified summaries on reasonable request.

**Figure 1.**
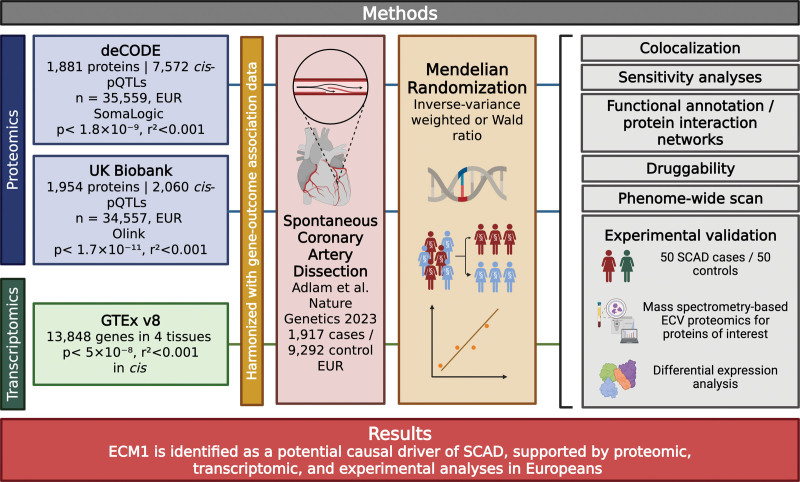
**Flowchart outlining methods and key findings.** ECV indicates extracellular vesicle; EUR, European; GTEx, Genotype-Tissue Expression; pQTLs, protein quantitative trait loci; and SCAD, spontaneous coronary artery dissection. Created in BioRender. Morley, A. (2025) https://biorender.com/hlsxmn3.

The flowchart outlining the design of the study is depicted in Figure 1. The full list and details of data sources used are provided in Table 1. The methods for this study can be found in the Supplemental Methods. Analyses of publicly available GWAS summary statistics did not require new ethics approval; ethics review and participant consent were obtained by the original studies.

**Table 1.**
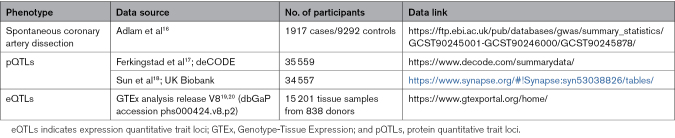
Details of Data Sources Utilized in This Study

The gene-outcome association estimates for all instruments leverage summary data of the GWAS meta-analysis by Adlam et al^16^ on 1917 cases with spontaneous coronary artery dissection (SCAD) and 9292 controls of European ancestry. The proteome-wide MR analysis uses gene-exposure association estimates, which influence protein levels (pQTLs) from 2 different cohorts based on different assays^21^; these cohorts include the Icelandic deCODE data set (n participants=35 559, n proteins=1881),^17^ using the aptamer-based SOMAscan^22^ v4, and the UKB (n participants=34 557, n proteins=1954),^18^ using the antibody-based Olink^23^ Explore 3072 platform. The transcriptome-wide MR analysis uses gene expression across arterial tissue (aortic, coronary, and tibial) and cultured fibroblasts in European participants of the GTEx consortium version 8.^19,20^ An in vitro experimental validation was conducted in a cohort of 50 patients with SCAD, and 50 healthy controls using mass spectrometry-based proteomic analysis of extracellular vesicles (ECVs). Participants were recruited from the UK Spontaneous Coronary Artery Dissection Registry and the SCAD Deep Phenotyping Study (ISRCTN42661582; UK National Research Ethics Service 14/EM/0056) and from the Leicester Biomedical Research Centre Cardiovascular Theme BRICCS study (UK National Research Ethics Service 09/H0406/114). Registry participants were enrolled across the UK via self-referral, primary care referral, or referral by the index-hospital clinical team. All participants provided fully informed signed consent.

## Results

### Proteome-Wide MR and Bayesian Colocalization Analysis

After instrument selection and harmonization with outcome association data in a European ancestry consortium of 1917 SCAD cases, and 9292 controls, 1264 of the 1881 proteins with cis-pQTLs in deCODE (using the aptamer-based SOMAscan v4), and 1322 of the 1954 proteins with cis-pQTLs in UKB (using the antibody-based Olink Explore 3072 platform) were available for analysis. All instruments exceeded the recommended F statistic threshold of 10, indicating no evidence of weak instruments. All instrument F statistics are presented in Table S1.

**Figure 2.**
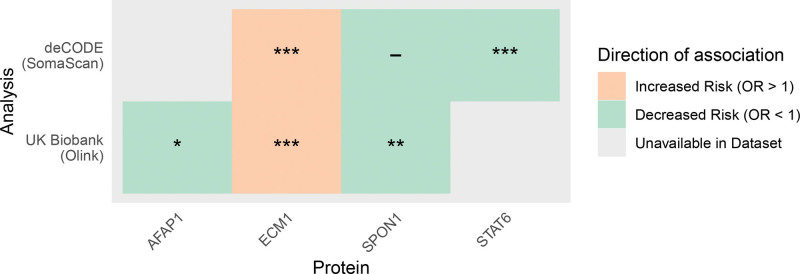
**Genetic associations of protein levels with spontaneous coronary artery dissection risk, displaying all proteins associated with spontaneous coronary artery dissection after correction for multiple testing in the main analysis.** Gray fields indicate data was not available for analysis. All *P* values are displayed after Benjamini-Hochberg correction for multiple testing. AFAP1 indicates actin filament–associated protein 1; ECM1, extracellular matrix protein 1; OR, odds ratio; SPON1, spondin 1; and STAT6, signal transducer and activator of transcription 6. *P<0.05; **P<0.01; and ***P<0.001.

Four proteins had significant genetic associations with SCAD (Table 2), reaching FDR-corrected statistical significance. Higher genetically predicted circulating levels of AFAP1 (actin filament–associated protein 1), SPON1 (spondin 1), and STAT6 (signal transducer and activator of transcription 6) were associated with lower genetically predicted risk of SCAD, suggesting protective effects of these proteins on SCAD risk (Figure 2; Tables 2 and 3). Though directionally consistent across data sets, the association of SPON1 was statistically significant in UKB but not in deCODE. Conversely, higher genetically predicted circulating levels of ECM1 (extracellular matrix protein 1) were associated with a greater genetically predicted risk of SCAD, suggesting detrimental effects of this protein on disease risk. Of those, only ECM1 and SPON1 had available *cis*-pQTLs across both deCODE and UKB. ECM1 had consistent associations with genetically predicted SCAD across both data sets, which were statistically significant, whereas SPON1 was only significant in UKB, despite consistency in direction of association, which was nominally significant before correction for multiple testing.

**Table 2.**
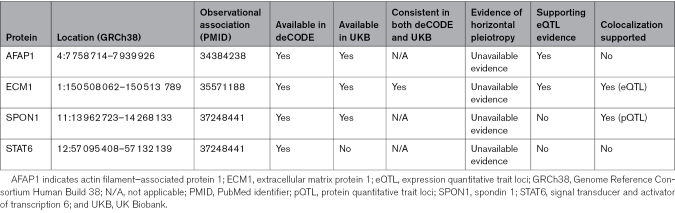
Summary of Proteome-Wide, Transcriptome-Wide, and Bayesian Genetic Colocalization Analysis for the Proteins Significant in the Main Analysis

### Sensitivity Analyses

For the pQTLs with significant associations with SCAD in the main analysis (ECM1, SPON1, AFAP1, and STAT6), MR using Egger regression (MREgger) and weighted median MR were not possible due to insufficient instruments at genome-wide significance level. For other pQTLs with sufficient available instruments, the results are reported in Tables S2 and S3.

### Colocalization

**Figure 3.**
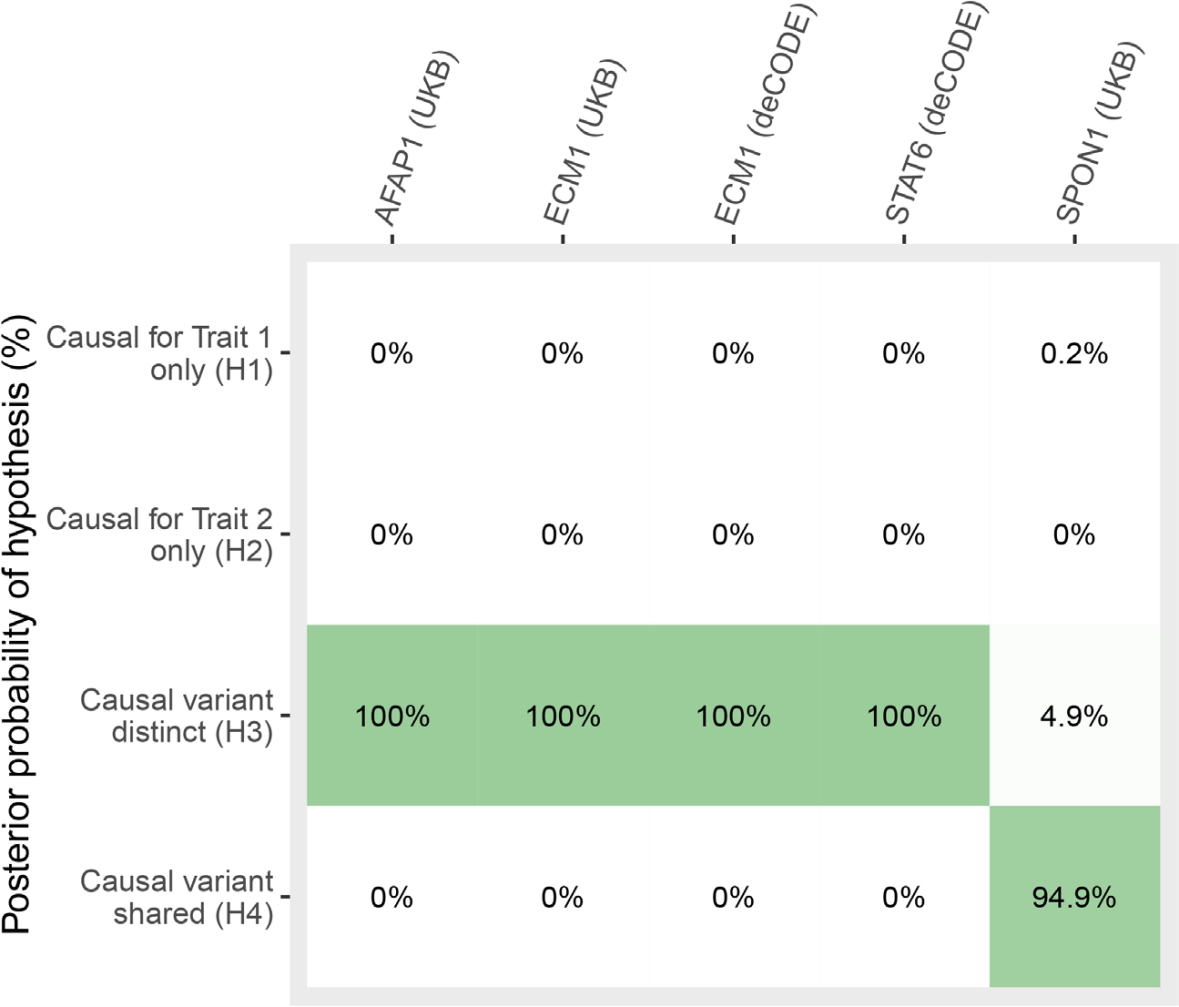
**Bayesian colocalization analysis evaluating the posterior probability of a shared causal variant influencing protein levels and spontaneous coronary artery dissection risk.** AFAP1 indicates actin filament–associated protein 1; ECM1, extracellular matrix protein 1; SPON1, spondin 1; and STAT6, signal transducer and activator of transcription 6; and UKB, UK Biobank.

Using Bayesian colocalization analyses, we evaluated the posterior probability of shared causal variants within protein-coding regions for the potentially causal pQTLs with SCAD (Tables S4 and S5). Only one of the prioritized proteins (SPON1) was supported by colocalization (Posterior Probability of Hypothesis 4, PP.H4=0.95) in the UKB. No colocalization was observed in deCODE, where only ECM1 was available among the prioritized proteins (Figure 3).

### Transcriptome-Wide MR and Bayesian Colocalization Analysis

**Figure 4.**
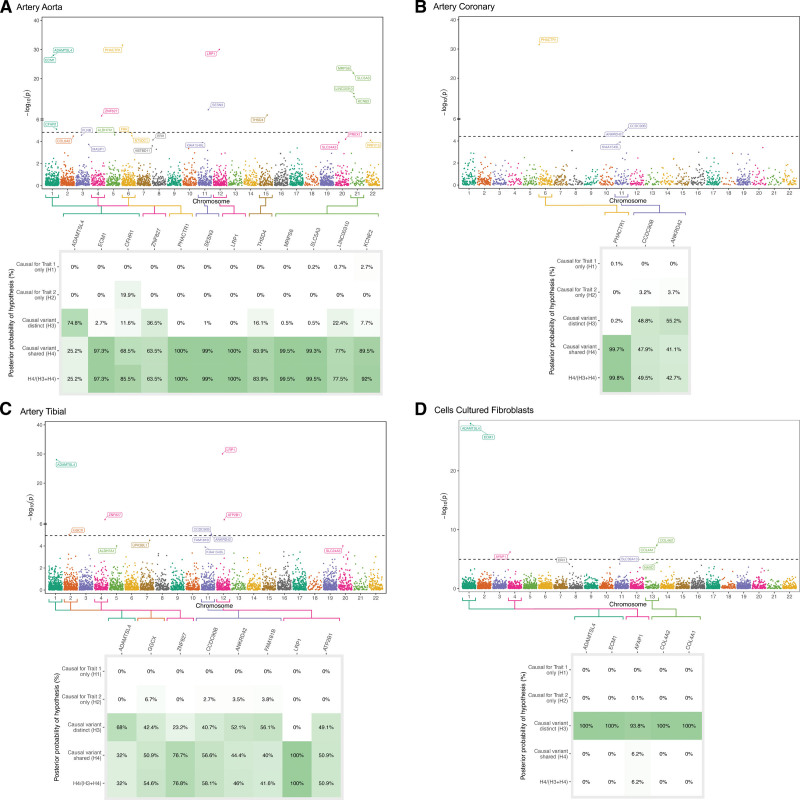
**Genetic association of gene expression levels with spontaneous coronary artery dissection, and corresponding Bayesian colocalization analysis results with probability of colocalization vs noncolocalization conditional on there being a causal variant for both traits (PP.H4/[PP.H3+PP.H4]).** Gray fields indicate data was not available for analysis. ADAMTSL4 indicates a disintegrin and metalloproteinase with thrombospondin motif-like protein 4; AFAP1, actin filament–associated protein 1; ANKRD42, ankyrin repeat domain 42; ATP2B1, plasma membrane calcium-transporting ATPase 1; CCDC90B, coiled-coil domain–containing protein 90B; CFHR1, complement factor H–related protein 1; COL4A1, collagen alpha-1(IV) chain; COL4A2, collagen alpha-2(IV) chain; ECM1, extracellular matrix protein 1; FAM181B, family with sequence similarity 181 member B; GGCX, vitamin K–dependent gamma-carboxylase; KCNE2, potassium voltage-gated channel subfamily E member 2; LINC00310, long intergenic nonprotein coding RNA 310; LRP1, low-density lipoprotein receptor–related protein 1 intracellular domain; MRPS6, mitochondrial ribosomal protein S6; PHACTR1, phosphatase and actin regulator 1; PP.H3, Posterior Probability of Hypothesis 3; PP.H4, Posterior Probability of Hypothesis 4; SESN3, sestrin-3; SLC5A3, solute carrier family 5 member 3; THSD4, thrombospondin type-1 domain–containing protein 4; and ZNF827, zinc finger protein 827.

In the transcriptome-wide MR, we evaluated the association of gene expression spanning >13 500 genes across arterial tissue (aortic, coronary, and tibial), as well as cultured fibroblasts, with SCAD. Overall, 20 expression quantitative trait loci (eQTLs) displayed at least 1 association with SCAD in at least 1 tissue that was significant after adjustment for multiple comparisons (Figure 4; Tables S6 and S7). Several of these are common to atherosclerotic coronary artery disease, including *COL4A1/COL4A2*, *LRP1*, *KCNE2*, *GGCX*, *MRPS6*, and *PHACTR1*. Comparing the eQTL results with those of the primary pQTL analysis, there was directionally consistent evidence corroborating the pQTL associations in at least 1 tissue for analysis for *AFAP1* and *ECM1*. Both were corroborated with cultured fibroblasts, and *ECM1* was also corroborated in aortic tissue. There was no evidence of a directionally consistent association for the remaining pQTLs. Of the significant pQTLs that were also significant in eQTL analysis, *ECM1* in aortic tissue was supported by colocalization (PP.H4=0.97), implying that variants that affect *ECM1* expression in aortic tissue also affect SCAD risk.

### Annotation

#### Protein Function, Interactions, and Druggability

The protein network displaying the 50 top high-confidence interactions (interaction score ≥0.70) for all 4 proteins is displayed in Figure S1. The closest interacting proteins, defined as those with a STRING interaction score of >0.90, with a maximum of 10 proteins (for each pQTL and eQTL) included, are provided in Table S8. The strongest STRING cluster was the JAK-STAT (Janus kinases and signal transducers and activators of transcription) signaling pathway, with STAT6 within this cluster and ECM1 distantly related via MMP9 (matrix metalloproteinase 9). Interleukin-2 family signaling and interleukins 4 and 13 were also distantly associated with STAT6 and ECM1. Full functional annotation for all proteins is provided in Table S9.

The results of the druggability evaluation for the significant pQTLs are presented in Table S10. All were considered druggable based on tractability information from OpenTargets, although none are a current target of clinically approved compounds or clinical-phase drug candidates.

#### Phenome-Wide Scanning

The results of phenome-wide scanning for all significant pQTLs are shown in Table S11. Among the 4 significant pQTLs, only ECM1 had genome-wide significant associations with other traits, including serum levels of TNFRSF13B (tumor necrosis factor receptor superfamily member 13B), genetically independent pain phenotypes (GIP1), and hip pain.

### Experimental Validation Analysis

**Figure 5.**
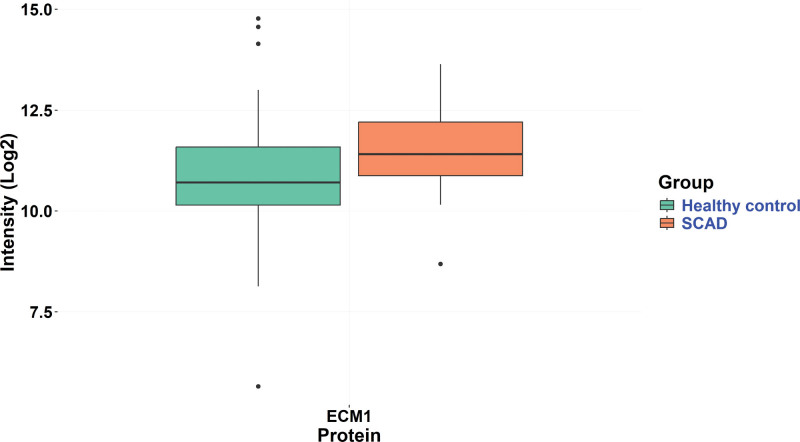
Boxplot of ECM1 (extracellular matrix protein 1) expression in extracellular vesicle convalescent plasma samples from patients with spontaneous coronary artery dissection (SCAD) and healthy controls.

Mass spectrometry-based proteomic analysis was performed on convalescent plasma ECV samples from a cohort comprising 50 patients with SCAD and 50 healthy controls (Figure 5). The demographic and clinical characteristics of individuals with SCAD (n=50) and healthy controls (n=50) are presented in Table 3. Mean age did not differ between groups (SCAD, 41.2±9.2 versus controls 44.0±8.6 years; *P*=0.116). All participants were female (100% in both groups, *P*=1.000). In addition, the ethnic distribution was similar, with 88% identifying as White British (*P*=0.230). Smoking status was also comparable with 64.0% of SCAD participants and 66.0% of controls being never smokers (*P*=0.977). Diabetes was present in 4.0% of patients with SCAD and none in the healthy controls, though this did not reach statistical significance (*P*=0.153). Hypertension was significantly more prevalent in the patients with SCAD (26.0%) as compared with healthy controls (2.0%; *P*<0.001). Dyslipidemia was also reported more frequently in the SCAD group (6.1%) versus none in the control group, but with a nonsignificant *P* value at 0.076. Among the candidate proteins of interest, ECM1 was the sole protein detected, while SPON1, AFAP1, and STAT6 were absent from the data set, falling below the detection threshold of our assay. Differential expression analysis revealed that ECM1 was significantly upregulated in the ECV SCAD samples with a log2-fold change of 0.633 (*P=*0.018) as compared with healthy controls.

**Table 3.**
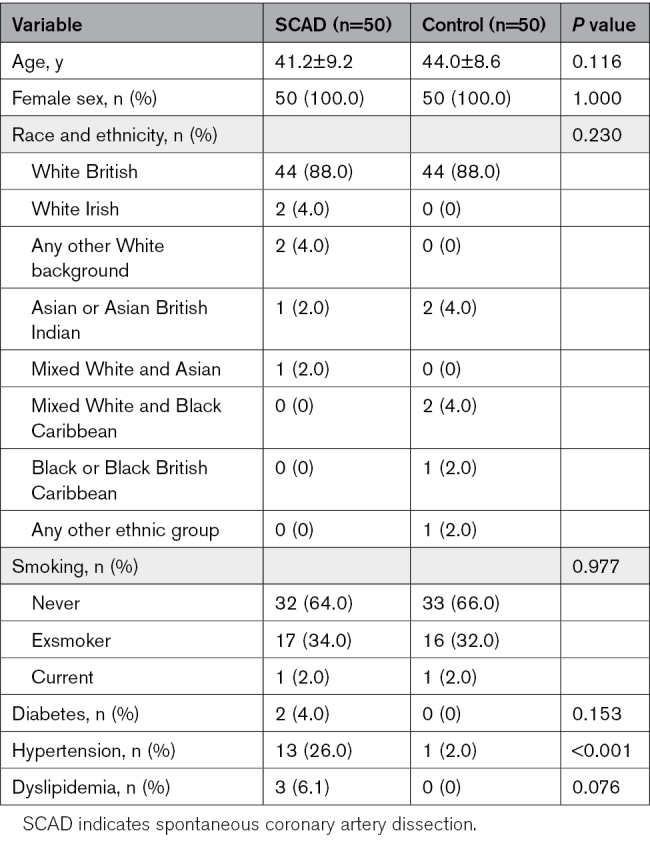
Patient Characteristics at Baseline in the Validation Cohort: Comparison Between the SCAD and Healthy Control Group

## Discussion

Using a multiomics approach, we aimed to identify potential biological pathways contributing to SCAD development. We identified 4 proteins associated with genetically predicted development of SCAD: AFAP1, ECM1, SPON1, and STAT6. Notably, ECM1 was significant among both sources for pQTL estimates, and this result was supported by colocalization analyses, suggesting a shared genetic basis of ECM1 levels and SCAD risk. JAK-STAT signaling, in addition to interleukin-2 signaling and interleukins 4 and 13, was identified as a potential biological pathway through annotation. Mass spectrometry proteomic analysis of ECV samples from patients with SCAD and controls supported the genetic associations and provided experimental evidence of the role of ECM1 expression in the pathogenesis of SCAD.

ECM1 is a glycoprotein involved in the development and maintenance of the ECM (extracellular matrix).^24^ Murine models have suggested this protein has a role in myocardial aging and infarction, with a suggestion of a profibrotic role.^25^ ECM1 is known to inhibit the activity of MMP9, a proteolytic enzyme, which regulates ECM remodeling.^26^ This reduction in ability to regulate the ECM could therefore leave vessel walls vulnerable to dissection, and we hypothesize that ECM1 contributes to a profibrotic environment within arterial vessels, as supported by the colocalization of ECM1 and SCAD within aortic tissue, which indicates that variants that affect ECM1 expression in aortic tissue also affect SCAD risk. This association was described in a previous, much smaller study of SCAD.^27^ We note that perturbation of ECM1 with reduced expression has been linked to hepatic fibrosis,^28^ mediated by ferroptosis with beneficial effects of therapeutic induction demonstrated in murine models.^29^ Therefore, strategies aimed at modulating ECM1 levels should consider potential systemic effects, including those on liver function. At the same time, further work will be required to clarify the biological underpinnings of this association. Notably, Encyclopedia of DNA Elements (ENCODE) based epigenetic analyses have suggested an inverse relationship,^16^ with the SCAD-associated variant linked to lower ECM1 chromatin accessibility. This finding highlights that the underlying mechanisms may be more complex than a simple causal link between increased ECM1 expression and SCAD risk. Overall, although ECM1 remains a compelling candidate locus, further investigation will be essential to reconcile these observations and to define its precise role in SCAD pathogenesis.

Increased STAT6 and AFAP1 expression was associated with lower risk of SCAD, suggesting a protective influence of anti-inflammatory states, or conversely, an element of unopposed inflammatory states contributing to SCAD pathophysiology. STAT6 has been described as pivotal to the anti-inflammatory role of interleukin-4^30^ and the ability of alternatively activated macrophages to inhibit T-cell proliferation.^31^ Systemic inflammation^32^ and periadventitial mixed inflammatory infiltrates, often with a predominance of eosinophils,^33^ have been reported in SCAD, although there were no significant observations of C-reactive protein, an acute phase inflammatory protein, which is consistent with our analysis, despite a small sample size.

In the proteome-wide analysis in UKB, higher levels of SPON1 were linked to a reduced risk of SCAD, a finding further supported by colocalization. However, this association was only significant in 1 of 2 proteomic data sets, although it was directionally consistent in both. Recent literature has identified a potential role of SPON1 in the development of other cardiovascular diseases, such as atrial fibrillation^34^; however, no previous studies have specifically examined the relationship between SPON1 levels and SCAD. SPON1 is a component of the ECM and is known to be expressed in arterial tissue, suggesting that its impact on SCAD may be similar to that of ECM1. Notably, SPON1 is also abundantly expressed in female reproductive tissues. Given the significantly higher incidence of SCAD in women, and its potential hormonal influences—highlighted by increased SCAD risk during pregnancy and postpartum—this connection may provide a clue to the underlying biological mechanisms.

Existing preclinical research and observational data align with the current understanding that SCAD is driven by inflammatory changes at the tissue level, coupled with alterations in ECM structure. In this study, identification of common protein clusters unique to SCAD, including the JAK-STAT pathway and interleukins 2, 4, and 13, which are critical to immune response regulation, further strengthens the inflammation-driven SCAD hypothesis. Although our findings contribute to this expanding body of evidence, several key factors must be considered before therapeutic approaches for SCAD can be pursued. Targeting proteins in disease requires (1) a clear understanding of the protein’s role in the disease, (2) the protein’s suitability for modulation by small molecules or biotherapeutics, and (3) a thorough evaluation of potential off-target effects. Given the consistent association between increased ECM1 expression and elevated SCAD risk in our analysis, along with current knowledge of phenotypic links, ECM1—and possibly SPON1—emerge as especially promising targets for further investigation.

### Limitations

Our study has several limitations. First, despite the use of large data sets in the analysis, the availability of exposure genetic variants remains limited. This has both reduced reproducibility of associations across the data sets, as well as the ability to undertake sensitivity analyses using MR-Egger and weighted median methods. Therefore, our capability to assess for potential horizontal pleiotropy was impeded in both the proteomic and transcriptomic analyses. We might have been able to include more single nucleotide polymorphisms (SNPs) had we decided to include proxies, however, this would lower the stringency of instrument selection from a pleiotropy risk perspective, and we therefore, opted not to do this. We further highlight that MR methods support causality, rather than confirm causality outright. Despite being the largest current available data set for SCAD, the sample size remains relatively small and, therefore, highlights systematic genetic investigation of patients with SCAD as a research priority. Restricting the population ancestry to European, to attenuate stratification which can violate the MR independence assumption, means our results may not be generalizable to individuals of other ancestries. Moreover, the reliability of genetic causal inference studies is dependent on meeting the instrumental variable assumptions of MR. As described in the methods, we use instruments that act in *cis* to reduce the risk of pleiotropy, and verified post hoc that instruments used for all proteins with significant associations were only associated with the protein of interest in *cis*, and not with other proteins. In addition to this, we restricted ancestry to reduce population stratification and corroborated our findings using gene expression data and colocalization. However, these results should be interpreted as supporting causality rather than establishing causality in isolation. Furthermore, the lack of colocalization of the ECM1 pQTL with SCAD risk may reflect a true lack of a causal variant or may be due to specific vulnerabilities of the platforms for epitope-binding artifacts, which can attenuate estimates. We also note that ECM1 eQTL expression in coronary arteries did not associate with SCAD risk and highlight that mRNA levels in single cells are often poor predictors of overall protein expression levels.^35^ This necessitates combined and integrative approaches, which, given the consistent results with ECM1 expression and SCAD risk over other proteomic, transcriptomic, and experimental analyses, support a causal role of ECM1 in SCAD pathogenesis. The mass spectrometry proteomic analysis was limited as ECM1 was the only protein available for analysis; moreover, the analysis was conducted on convalescent plasma samples, which despite providing evidence for dysregulation of ECM1 in SCAD pathogenesis, is unable to provide evidence for a temporal causal association of ECM1 driving the disease process. It is worth noting that some prior studies have reported apparently opposing directions of association at loci shared between SCAD and atherosclerotic coronary artery disease. Our analysis was designed to focus specifically on biological mechanisms underlying SCAD, and we therefore did not undertake a systematic comparison of directionality across traits. Nonetheless, such observations highlight an important area for future work. Finally, as most instruments used for this high-throughput analysis included single SNPs, this work should be interpreted as hypothesis-generating, rather than definitive findings. We therefore highlight that further triangulation of investigational approaches is necessary to establish causality, as this cannot be solely established by genetic studies.

### Conclusions

Through an integrative analysis of large-scale proteomic and transcriptomic data, we have identified 4 circulating proteins that show both functional significance and a potential causal link to genetically proxied SCAD risk. Among these, ECM1 stands out as a key protein, supported by consistent findings across proteomic data sets, transcriptomic evidence from aortic tissue, and ECV proteomics of convalescent plasma samples from patients with SCAD. Although SCAD is relatively uncommon, it disproportionally affects younger women, often during the peripartum period, resulting in a significant health burden and underscoring the urgent need for disease-modifying treatments. The findings of this study support a causal role for ECM1, and possibly SPON1, in SCAD pathophysiology, and these proteins should be prioritized in future mechanistic studies and as potential pharmaceutical treatment targets.

## Article Information

### Acknowledgments

The authors acknowledge all investigators and participants of the studies contributing to the present analyses. The views expressed are those of the authors and not necessarily those of the National Institute for Health and Care Research or the Department of Health and Social Care. The Graphical Abstract was created in BioRender. Morley, A. (2025) https://BioRender.com/hlsxmn3. License to use the BioRender content, including icons, templates, and other original artwork was granted to A.P. Morley (Agreement number UT29BB0FEO). The mass spectrometry work was supported by the John and Lucille van Geest Foundation and the National Institute for Health and Care Research, Leicester Biomedical Research Centre.

### Sources of Funding

M. Ardissino is supported by a Medical Research Council Clinical Research Training Fellowship (MR/Z505146/1). R.K. Reddy is supported by the Nuffield Department of Population Health, University of Oxford. B.P. Halliday is supported by the British Heart Foundation (FS/ICRF/24/26128) and Rosetrees Trust. T.H. Cao, P.A. Quinn, L.L. Ng, A.A. Baranowska-Clarke, T. Webb, and D. Adlam are supported by the Leicester National Institute for Health and Care Research Biomedical Research Centre, United Kingdom. L.L. Ng has received grants from the John and Lucille Van Geest Foundation and National Institute for Health and Care Research. P. Natarajan is supported by the US National Heart, Lung, and Blood Institute and the US National Human Genome Research Institute (NHLBI R01HL127564, NHGRI U01HG011719). A.S. Butterworth is supported by core funding from the British Heart Foundation (RG/18/13/33946) and the National Institute for Health and Care Research Cambridge Biomedical Research Centre (BRC-1215-20014; NIHR203312). This work was supported by additional core funding from the Cambridge British Heart Foundation Centre of Research Excellence (RE/18/1/34212) and the British Heart Foundation Chair Award (CH/12/2/29428). M.C. Honigberg is supported by the US National Heart, Lung, and Blood Institute (NHLBI, R01HL173028) and the American Heart Association (24RGRSG1275749, 25SFRNCCKMS1443062, and 25SFRNPCKMS1463898). D. Adlam is funded by BeatSCAD and the British Heart Foundation (SP/F/24/150073). A. de Marvao is supported by the Fetal Medicine Foundation (495237).

### Disclosures

B.P. Halliday has been a consultant for AstraZeneca and Zoll. P. Natarajan reports research grants from Allelica, Amgen, Apple, Boston Scientific, Genentech/Roche, and Novartis, personal fees from Allelica, Apple, AstraZeneca, Blackstone Life Sciences, Bristol Myers Squibb, Creative Education Concepts, CRISPR Therapeutics, Eli Lilly & Co, Esperion Therapeutics, Foresite Capital, Foresite Labs, Genentech/ Roche, GV, HeartFlow, Magnet Biomedicine, Merck, Novartis, Novo Nordisk, TenSixteen Bio, and Tourmaline Bio, equity in Bolt, Candela, Mercury, MyOme, Parameter Health, Preciseli, and TenSixteen Bio, and spousal employment at Vertex Pharmaceuticals, all unrelated to the present work. A.A. Baranowska-Clarke reports institutional grants from AstraZeneca, Bayer, Biogen, BioMarin, Bioverativ, Novartis, Regeneron, and Sanofi. Dr Honigberg reports consulting fees from Comanche Biopharma, grant support from Genentech, and site principal investigator work for Novartis, all unrelated to the present work. D. Adlam has received funding to support a clinical research fellow from Abbott Vascular. He has also received in-kind support from AstraZeneca, Inc to support gene sequencing in spontaneous coronary artery dissection and has conducted unrelated consultancy for GE Inc. He holds patents for medical devices, including a cardiac assist device (EP3277337A1, PCT/GB2017/050877, and UK PATENT APPLICATION NUMBER 2211616.4). He is chief medical officer and founder of Intervent Cardio Ltd focused on commercialization of these devices. He receives royalties from Elsevier Inc. for ECG Made Easy, ECG Made Practical, and ECG Problems Books. The other authors report no conflicts.

### Supplemental Material

Supplemental Methods

Tables S1–S11

Figure S1

References 36–57

## Supplementary Material


