# Supplementary material for: Genetic Association of Circulating Proteins and Gene Transcripts With Spontaneous Coronary Artery Dissection
Source: Circ Genom Precis Med. 2026 Jan 29;19(1):e005330. doi: 10.1161/CIRCGEN.125.005330 (PMC12915550; doi:10.1161/CIRCGEN.125.005330)

Supplemental Methods

This study uses publicly available GWAS summary data which had ethical approval and full participant consent according to the protocols available at the cited sources[16-20]. The flowchart outlining the design of the study is depicted in **Figure 1**. The full list and details of data sources used is provided in **Table 1**. Analyses were carried out on R version 4.4.1[36] using the TwoSampleMR (version 0.6.4)[37] and Mendelianrandomization (version 0.10.0)[38] packages.

Proteome-wide Mendelian Randomization

Instrumental variant selection

We first performed a proteome-wide MR analysis using genetic variants which influence circulating protein levels in plasma (protein quantitative trait loci, pQTLs) across two different cohorts which utilize different assays[21]; these cohorts included the Icelandic deCODE dataset (n participants = 35,559, n proteins = 1,881)[17], using the aptamer-based SOMAscan[22] v4, and the UK Biobank (UKB, n participants = 34,557, n proteins = 1,954)[18], using the antibody-based Olink[23] Explore 3072 platform. Selection of two different cohorts for the purpose of the analysis helps to attenuate potential false discoveries because of specific limitations of each individual assay. We extracted uncorrelated ($r^2 < 0.001$) single nucleotide polymorphisms (SNPs) associated with the proteins at genome-wide significance level accounting for the number of proteins considered ($P < 1.8 \times 10^{-9}$ in deCODE and $P < 1.7 \times 10^{-11}$ in UKB) and acting in *cis* (within 1Mb of the protein-encoding gene region) for analysis. The linkage disequilibrium pruning strategies and allele frequency thresholds for the *cis*-pQTLs are available in the original publications[17,18].

Outcome data

The gene-outcome association estimates for all instruments were extracted from summary data of Adlam et al.'s GWAS meta-analysis on 1,917 cases with spontaneous coronary

artery dissection (SCAD) and 9,292 controls of European ancestry[16]. We retained SNPs with a corresponding association estimate in the SCAD GWAS meta-analysis; unmatched SNPs were discarded, and no proxies were sought. Harmonization of gene-exposure and gene-outcome association data was performed using the harmonize_data function in TwoSampleMR[37]. During harmonization, where possible strand direction was inferred, and palindromic SNPs were harmonized. If this was not possible due to incompatible or ambiguous alleles, the SNP was eliminated from further analysis.

Statistical analysis

Inverse variance-weighted MR[39] with fixed-effects was performed to estimate the association between genetically-predicted pQTL and SCAD, where more than one instrumental variable was present. In the case of a single instrumental variable, the Wald ratio[40] method was used. Within the analyses, an expected 5% false discovery rate (FDR) was controlled for using Benjamini-Hochberg[41] correction of *P*-values. Results for the primary analyses are presented as odds ratios (ORs) and 95% confidence intervals (95% CI) for every 1 standard deviation (1-SD) higher genetically-predicted protein level, alongside FDR-adjusted *P*-values. In the main analysis, we considered FDR-adjusted *P*-values <0.05 as statistically significant.

Sensitivity analyses

For the results of MR analysis to be valid, instruments must satisfy three key assumptions[39]:

(1) Relevance: that there is association of variants with the exposure.
(2) Independence: that there exists no unmeasured common causes of the genetic variant and the outcome. Within MR, this may occur if there is significant population stratification, which can occur when populations of different ancestry are used in the same MR analysis.
(3) Exclusion restriction: the variant only influences the outcome through the exposure, and not through alternative pathways.

The first assumption was verified through calculation of the instrument F-statistics using the formula:

$$F = \frac{(n-k-1)}{k}\frac{(R^2)}{(1-R^2)}$$

where R^2 is the explained variance in the regression of all SNPs, n is the number of participants in the study, k is the number of instrumental variants. The R^2 was calculated as the sum of SNP-wise R^2 of instruments, which is obtained as follows:

$$R^2 = \frac{F}{(n-2+F)} \text{ with } F = \left(\frac{\beta}{SE(\beta)}\right)^2$$

where β represents the effect size of the genetic variant in the exposure GWAS, and $SE(\beta)$ represents the standard error of the effect size of the genetic variant in the exposure GWAS. Generally, F-statistics <10 indicate the presence of weak instruments which can result in weak instrument bias[42]. The second assumption may be violated due to population stratification; this was mitigated by selection of data sources for both gene-exposure and gene-outcome associations from studies that (1) adjusted for principal components (age and sex) and (2) from single ancestry populations (in this case, European ancestry). The third assumption cannot be formally tested but was evaluated through sensitivity analyses using MR-Egger[43] and weighted median MR[44] where this was possible (where ≥3 instrumental variables were available). We also restricted analyses by using SNPs acting in *cis* (within 1Mb of the protein-encoding gene region), which further mitigates pleiotropy[45,46].

Transcriptome-wide Mendelian randomization

To further the analysis, we explored the role of gene expression across arterial tissue (aortic [n= 387], coronary [n=213], and tibial [n=584]), as well as cultured fibroblasts (n=855) with SCAD risk in European participants of the Genotype-Tissue Expression (GTEx) consortium version 8[19,20]. Genome-wide significant ($P <5\times10^{-8}$), uncorrelated ($r^2 <0.001$) variants acting in *cis* (within 1Mb of gene region) for tissue-specific eQTLs (expression quantitative trait loci)

were extracted. We then proceeded with harmonization and analysis utilizing the same methodology as the proteomic analysis.

Bayesian colocalization analysis

Bayesian colocalization analyses can be used to evaluate whether two traits (e.g. circulating levels of a protein and a disease outcome) are driven by a shared causal variant within a genetic region. Evidence of colocalization supports the notion that the two traits under investigation share a causal association. More specifically, Bayesian colocalization analyses[47] for any genetically-predicted exposure and outcome pair can be used to assess the posterior probability of genetic variants within a specific gene region having:

- No association with either exposure or outcome (PP.H0)
- Association with exposure, not with outcome (PP.H1)
- Association with outcome, not with exposure (PP.H2)
- Association with exposure and outcome, two distinct causal SNPs (PP.H3)
- Association with exposure and outcome, both due to a single causal SNP (PP.H4)

A PP.H4 of ≥80% can be interpreted as strong colocalization evidence. We further calculated the PP.H4 / (PP.H3+PP.H4) value; this is the probability of colocalization conditional on presence of a causal variant for the outcome. This is particularly useful when PP.H1 or PP.H2 are high, as it can help to discern if remaining evidence favors colocalization or non-colocalization[48]. We performed these analyses for all proteins with a statistically significant association in the main MR analysis. For the colocalization analysis, significant instruments for *cis*-pQTLs (acting within 1Mb of the protein-encoding gene region) were extracted from deCODE[17] and UKB[18] data. Corresponding variants (in the same position) were then extracted from the SCAD[16] GWAS meta-analysis data. Analyses were performed using coloc R package[47,49,50] v5.2.223 with prior probabilities set as $p_1=1\times10^{-4}$, $p_2=1\times10^{-4}$, and $p_{12}=1\times10^{-5}$.

Network-Based Approaches and Annotation

Protein function, interactions and druggability

Initially the STRING protein interaction browser[51] was utilized by selecting the significant pQTLs and SCAD risk genetic associations from the proteomic MR analysis, extracting the 50 top proteins with functional and physical interactions within the network, with minimum interaction score of 0.70. The STRING interaction network portal[51] was then used to annotate proteins with significant pQTL / eQTL and SCAD risk genetic associations. We then assessed druggability with OpenTargets[52] and current research of the druggable genome[53].

Phenome-wide scan

To evaluate predicted effects of protein level modulation and thus the potential impacts of pharmaceutical intervention, phenome-wide scans (PWS) were performed using the NHGRI-EBI GWAS Catalog[54] with a significance threshold of $P < 5\times10^{-8}$.

Experimental validation analysis

To validate the results of the genetics-based inferential approach, we performed a mass spectrometry-based proteomic analysis on a cohort comprising 50 SCAD patients and 50 healthy controls. Convalescent plasma samples were collected, and proteins associated with extracellular vesicles (ECV) were isolated and then analyzed using liquid chromatography electrospray ionisation tandem mass spectrometry (LC-ESI-MS/MS). We specifically examined the differential expression of candidate proteins of interest from the genetic analysis.

Sample preparation

A 200 µL plasma sample was pre-cleared by centrifugation at 3000 g for 10 minutes. Following this, 22.2 µL of 1 M ammonium acetate was added, mixed, and incubated on ice for 45 minutes. Next, 1200 µL of 100 mM ammonium acetate was introduced, and the mixture was centrifuged at 20000 g for 30 minutes. Proteins associated with ECVs were

retained on the side walls of the Eppendorf for downstream analysis. Subsequently, 100 µL of a premix containing ammonium deoxycholate (ADC), DL-dithiothreitol (DTT), and ammonium bicarbonate (formulated as ADC 2%, 50 mM ammonium bicarbonate, and 1 M DTT) was added to reduce disulphide bonds, with incubation at 65 °C for 30 minutes. The reduction step was followed by alkylation using 20 mM iodoacetamide (IAA) for 30 minutes in the dark. The sample volume was then adjusted using 50 mM ammonium bicarbonate to achieve optimal pH conditions for enzymatic digestion. Trypsin was added at a 1:25 enzyme-to-protein ratio, and digestion was carried out overnight at 37 °C. Peptides were then extracted using solid-phase EMPORE C18 discs and eluted sequentially with 60% acetonitrile (ACN) containing 0.1% formic acid (FA) followed by 80% ACN with 0.1% FA. The eluate was subjected to speed vacuum for 90 minutes to remove ACN, then freeze-dried overnight. The dried peptides were resuspended in 0.1% FA, and their concentrations were quantified using a peptide assay. An internal standard (yeast alcohol dehydrogenase, ADH) was spiked into each sample, with 100 fmol injected per run.

Liquid chromatography electrospray ionisation mass spectrometry/mass spectrometry analysis (LC-ESI-MS/MS)

A Waters Synapt G2S High Definition Mass Spectrometry (HDMS) system (Waters Corporation, Manchester, UK) which features a nano electrospray ionisation (ESI) source, stepwave ion guide, quadrupole, triwave, and time-of-flight (TOF) analyser was employed to analyse the samples. The system was coupled to a nanoAcquity UPLC setup, incorporating a C18 trapping column (180 µm × 20 mm, 5 µm) for desalting and contaminant removal, along with an HSS T3 analytical column (75 µm × 150 mm, 1.8 µm) directly connected to a NanoLockSpray ESI interface. Samples were introduced at a flow rate of 0.3 µL/minute using an emitter. The mobile phases were composed of solvent A (0.1% formic acid in HPLC-grade water) and solvent B (0.1% formic acid in acetonitrile). The analytical method employed ion mobility-enabled, data-independent acquisition (IM-LC-DIA-MS). For each injection, 1 µg of ECV samples were loaded, with the analysis sequence randomized to

reduce batch effects. Each sample was analyzed using a 110 minute gradient in HDMSE mode.

Proteomic data processing and analysis

Proteomic data processing was conducted using Progenesis QI for Proteomics version 4.2 (Nonlinear Dynamics, Waters Corporation, UK) for the identification and quantification of peptides and proteins. A human protein database in FASTA format was retrieved from UniProtKB and utilized for matching spectral data to peptide and protein entries. The protein mass limit was set to a maximum of 1000 kDa. Digestion parameters followed strict trypsin cleavage rules that allows up to two missed cleavages. Carbamidomethyl C was specified as a fixed modification, while Deamidation N, Oxidation M were included as variable modifications. For peptide and protein identification, thresholds included a minimum of 3 fragment ions per peptide, 7 fragments per protein, and at least 2 peptides per protein. The false discovery rate (FDR) was controlled at less than 1%. Relative protein quantification was based on the Hi-3 method that uses the three most abundant peptides per protein. Output from Progenesis was exported to Microsoft Excel for further data analysis. The data were denoised using the NOISeq R package[55,56] and subsequently log2-transformed for downstream analysis. LIMMA (Linear Models for Microarray Data) R package was employed to perform differential expression analysis for the proteomic data[57].

Supplemental Legends

Supplemental Table 1 – F-statistic for all instrument sets used in the study calculated from the respective exposure GWAS. SNP = single nucleotide polymorphism.

Supplemental Table 2 – Genetic associations of protein levels with spontaneous coronary artery dissection, using instruments acting in *cis* and extracted from the deCODE study. SNP = single nucleotide polymorphism.

Supplemental Table 3 – Genetic associations of protein levels with spontaneous coronary artery dissection, using instruments acting in *cis* and extracted from the UK Biobank study. SNP = single nucleotide polymorphism.

Supplemental Table 4 – Bayesian colocalization analysis evaluating the posterior probability of a shared causal variant influencing protein levels in deCODE and spontaneous coronary artery dissection risk. PP.H0 = posterior probability of hypothesis 0 (no causal variants); PP.H1 = posterior probability of hypothesis 1 (causal variant for trait 1 only); PP.H2 = posterior probability of hypothesis 2 (causal variant for trait 2 only); PP.H3 = posterior probability of hypothesis 3 (distinct causal variants); PP.H4 = posterior probability of hypothesis 4 (shared causal variant); SNP = single nucleotide polymorphism.

Supplemental Table 5 – Bayesian colocalization analysis evaluating the posterior probability of a shared causal variant influencing protein levels in UK Biobank and spontaneous coronary artery dissection risk. PP.H0 = posterior probability of hypothesis 0 (no causal variants); PP.H1 = posterior probability of hypothesis 1 (causal variant for trait 1 only); PP.H2 = posterior probability of hypothesis 2 (causal variant for trait 2 only); PP.H3 = posterior probability of hypothesis 3 (distinct causal variants); PP.H4 = posterior probability of hypothesis 4 (shared causal variant); SNP = single nucleotide polymorphism.

Supplemental Table 6 – Genetic associations of tissue-specific gene expression levels with spontaneous coronary artery dissection. SNP = single nucleotide polymorphism.

Supplemental Table 7 – Bayesian colocalization analysis evaluating the posterior probability of a shared causal variant influencing tissue-specific gene expression levels and spontaneous coronary artery dissection risk. PP.H0 = posterior probability of hypothesis 0 (no causal variants); PP.H1 = posterior probability of hypothesis 1 (causal variant for trait 1 only); PP.H2 = posterior probability of hypothesis 2 (causal variant for trait 2 only); PP.H3 = posterior probability of hypothesis 3 (distinct causal variants); PP.H4 = posterior probability of hypothesis 4 (shared causal variant); PP.H4 / (PP.H3+PP.H4) = probability of colocalization versus non-colocalization conditional on there being a causal variant for both traits; SNP = single nucleotide polymorphism.

Supplemental Table 8 – Summary of evidence supporting the 10 top functional and physical interactions of proteins, only including those with STRING interaction score ≥0.90.

Supplemental Table 9 – Functional annotation of significant proteins.

Supplemental Table 10 – Druggability of the proteins identified as potential therapeutic targets for spontaneous coronary artery dissection. PROTAC = proteolysis targeting chimera.

Supplemental Table 11 – Summary of phenome-wide scan evaluating potential on-target effects associated with intervention on the identified proteins. UKB = UK Biobank; SNP = single nucleotide polymorphism.

Supplemental Figure 1 – Protein interaction network displaying the 50 top interacting proteins in the network with STRING interaction score ≥0.70.

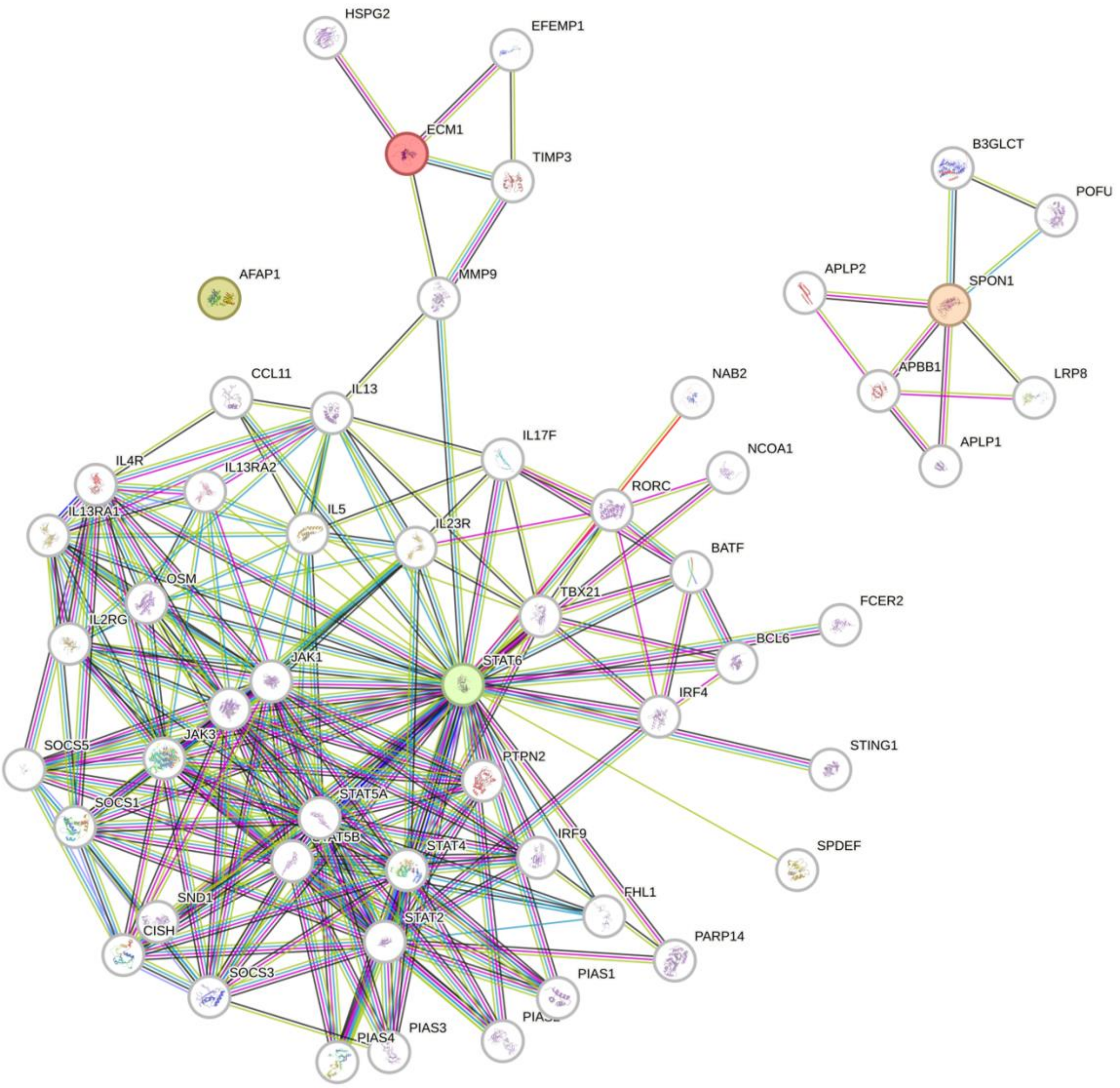

Supplement: Supplementary file 1 [file hcg-19-e005330-s001.pdf]
